# Development and Utilization of a Model System to Evaluate the Potential of Surface Coatings for Protecting Grapes from Volatile Phenols Implicated in Smoke Taint

**DOI:** 10.3390/molecules26175197

**Published:** 2021-08-27

**Authors:** Julie A. Culbert, Mark P. Krstic, Markus J. Herderich

**Affiliations:** The Australian Wine Research Institute, P.O. Box 197, Glen Osmond, SA 5064, Australia; mark.krstic@awri.com.au (M.P.K.); markus.herderich@awri.com.au (M.J.H.)

**Keywords:** surface coatings, agrochemicals, horticultural products, volatile phenols, glycosides, smoke taint, grapes

## Abstract

Due to the increasing frequency of wildfires in recent years, there is a strong need for developing mitigation strategies to manage the impact of smoke exposure of vines and occurrence of ‘smoke taint’ in wine. One plausible approach would be to prevent or inhibit the uptake of volatile phenols from smoke into grape berries in the vineyard. In this study we describe a model system we developed for evaluating under controlled conditions the effectiveness of a range of surface coatings (including existing horticultural sprays) for reducing/preventing the uptake of volatile phenols and their subsequent conversion to phenolic glycosides. Grapes were coated with the materials to be tested and then exposed to gaseous phenols, via evaporation from an aqueous solution, in a semi-closed glass container. Analysis of volatile phenols and their glycosidic grape metabolites demonstrated that the treatments typically did not provide any significant protection; in fact, some resulted in higher concentrations of these compounds in the grapes. The highest concentrations of volatile phenols and their glycosides were observed after application of oily, hydrophobic materials, suggesting that these materials may enhance the adsorption or transfer of volatile phenols into grape berries. Therefore, it is important to consider the types of sprays that are being applied in the vineyard before and during smoke events to prevent the potential of exacerbating the uptake of smoke compounds by grape berries.

## 1. Introduction

The presence of smoke in vineyards, from wildfires or prescribed burns, is of major concern to viticulturists and winemakers as grapevines are susceptible to the uptake of volatile phenols contained in smoke, such as guaiacol, isomeric cresols and syringols [1,2,3,4]. Once absorbed into the berry, these phenols are enzymatically conjugated to sugars; typically, a glucose unit is added first followed by a second sugar (pentose or hexose) leading to disaccharide formation [5] More recent studies have shown that trisaccharides can also be formed [6]. During winemaking, volatile phenols can be released into the wine by hydrolysis of the sugars from these non-volatile phenolic glycosides, therefore contributing to ‘smoky’ and ‘ashy’ aromas and flavors [1,6,7]. In addition, volatile phenols can be released in-mouth by enzymatic cleavage of phenolic glycosides through glycosidases present in saliva and oral microbiota [8,9]. Retronasal perception of volatile phenols released in-mouth creates a lingering ‘smoky’ and ‘ashy’ aftertaste [10]. These negative sensory characteristics in wine caused by smoke exposure of grapes represent an undesirable quality defect in wine, commonly referred to as ‘smoke taint’.

Given the increased incidence of bushfires and wildfires impacting the wine industry globally, there is a growing need to mitigate the effects of smoke exposure of vines. Mitigation strategies evaluated to date have been summarized in two review papers [11,12]. The major strategies involve either preventing or inhibiting the uptake of volatile phenols from smoke into the grape berry in the vineyard and detection of ‘clean’ grapes and separating ‘clean’ from smoke-affected grapes at harvest. These vineyard-based approaches are typically combined with winery management practices aiming to reduce the extraction of smoke taint compounds into must and/or to remediate smoke affected grape juice and wine via subtractive treatments. While the prevention of uptake of smoke molecules in the vineyard is a more attractive approach, as prevention is better than cure, there have been limited studies and no substantive success reported in this space thus far. Application of misting via an in-canopy sprinkler system by Szeto and colleagues (2020) partially reduced the uptake of volatile phenols by grapes during smoke exposure of vines, but not to a level required for significantly reducing the sensory perception of smoke taint in wine [13]. Other studies have focused on the ability of kaolin clay [14,15] or biofilm [16], applied to grapevines, to inhibit the uptake of smoke taint molecules during smoke exposure. The results from these studies showed some promise, but also highlighted the need for further research as treatments were not effective for all varieties evaluated [14] or the timing and efficacy of spray application prior to a smoke exposure incident warrants further optimization [16].

The application of horticultural products (and agrochemicals) to grapevines is common practice in viticulture to negate the quality and economic impacts of pests and disease [17,18,19]. For instance, fungal diseases such as powdery mildew, downy mildew and botrytis rot can be controlled by application of fungicides [20,21]. In more recent years, there has been an increase in the application of sunscreen protectants for the protection of crops against extreme heat [22]. The application of chemical reflectants (i.e., kaolin, calcium carbonate) to fruit can reduce sunburn severity by reflecting UV and IR light [23,24,25].

Smoke taint research in wine grape vineyards is challenging because of the relatively short ripening period of grapes along with the inherent unpredictable occurrence and danger of bushfires. This necessitates the use of model systems such as purpose-built smoke tents where grapevines can be exposed to smoke generated from the combustion of model fuels such as straw [1,26,27,28]. While smoke tents allow for controlled and repeatable experiments, there are limitations with regard to precisely controlling the smoke composition and number of grapevines, and hence, quantity of fruit, that can be exposed to model smoke at one time. Exposing excised grape bunches to smoke post-harvest, could overcome this limitation and Kennison and colleagues (2007) showed that wine with elevated levels of smoke-derived volatile phenols could be produced via this method [26]. However, until now it was unknown whether post-harvest smoke exposure of grapes would result in the in vivo glycosylation of volatile phenols as the existence of phenolic glycosides had not been established at the time of that previous study. Other model studies have shown that grapes and leaves can absorb guaiacol after application of aqueous solutions of phenolic substrates [2]. Similarly, foliar applications of guaiacol or oak extracts to Monastrell grapevines resulted in the accumulation of guaiacol glycoconjugates [29]. These observations highlight the suitability and ease of using aqueous solutions of volatile phenols as an alternative to generating smoke from the combustion of fuel.

The aims of this study were to (i) establish a model system for studying the consequences of exposure of grape bunches to volatile phenols; (ii) investigate if excised grape bunches can absorb gaseous volatile phenols generated by their evaporation from an aqueous mixture and if they will subsequently convert them to phenolic glycosides; and (iii) utilize this model system to evaluate a range of horticultural surface coating products currently permitted for use by Australian growers, and other materials, for their potential to reduce or prevent the uptake of volatile phenols by grape berries and their subsequent conversion to phenolic glycosides.

## 2. Results and Discussion

### 2.1. Absorption of Gaseous Phenols by Excised Grape Bunches—Proof-of-Concept Experiments

Initial experiments focused on whether excised grape bunches (i.e., those removed from the vine) are able to absorb volatile phenols and convert them to phenolic glycosides. Previous work by Kennison et al. (2007) had demonstrated that post-harvest smoke exposure of whole Verdelho grape bunches resulted in smoke-affected wine [26]. The presence of guaiacol, 4-methylguaiacol, 4-ethylguaiacol, 4-ethylphenol, eugenol and furfural in these wines was attributed to the smoke exposure. However, this work was performed prior to researchers identifying the presence and importance of the phenolic glycosides in smoke-exposed fruit [3]. Hence, at the time, phenolic glycosides were not measured and no data were available about the activity of glucosyl transferases and formation of phenolic glycosides in excised grapes exposed to post-harvest smoke. Therefore, the first key question to be addressed was whether grape enzymes responsible for conversion of volatile phenols to phenolic glycosides (i.e., glycosidases) are active in post-harvest excised grape bunches. In order to evaluate this, grape bunches were placed inside a glass vessel and exposed to gaseous volatile phenols released by evaporation from an aqueous substrate solution consisting of five phenols typically present in smoke, guaiacol, *o*-cresol, *m*-cresol, syringol and phenol. Following exposure, the concentrations of volatile phenols and phenolic glycosides in grape homogenates were quantified and compared to those of control grape bunches stored in the absence of volatile phenols. Due to their availability all year round, initial experiments were performed with white seedless table grapes (variety unknown) and confirmed by model studies involving field-grown wine grapes once mature grapes became available close to harvest.

#### 2.1.1. Table Grapes

After white seedless table grape bunches had been exposed to volatile phenols for approximately 2.5 days, volatile phenols and phenolic glycosides were quantified with the GC-MS and LC-MS methods used routinely for detecting smoke exposure of grapes. As expected, the results in Table 1 confirm that the untreated control table grapes did not contain smoke volatile phenols and phenolic glycosides above the analytical limits of detection. Clearly elevated concentrations were observed in the grapes exposed to gaseous phenols. These results established that volatile phenols evaporated from an aqueous solution can be absorbed by table grapes and within hours converted to phenolic glycosides. The most abundant glycosides observed were the pentosylglucosides and monoglucosides of guaiacol and *o*-cresol (i.e., GuPG, CrPG, GuMG and CrMG) (Table 1).

In contrast to what has been observed for grapes collected in vineyards sometime after smoke exposure from bushfires [30], the concentrations of volatile phenols in the grapes from these model experiments were still relatively high. This most likely reflects the continuous exposure of excised bunches to volatile phenols until the model exposure treatment was ended, with not enough time between exposure and homogenization of grapes for complete conversion of the free phenols to their glycosidic metabolites. In the grape homogenates, higher concentrations of *o*-cresol and guaiacol were observed compared to *m*-cresol and syringol, which likely reflects the higher volatility and faster evaporation of these phenols from the aqueous solution.

#### 2.1.2. Wine Grapes

To validate the initial study with table grapes, the experiments were repeated with excised bunches of Chardonnay, Sauvignon Blanc and Semillon grapes during the 2017 harvest. Concentrations of the phenolic glycosides in the control and treated Chardonnay, Sauvignon Blanc and Semillon grapes are given in Table 2. The concentrations of volatile phenols in these wine grapes were not measured as the presence of the phenolic glycosides was indicative that volatile phenols had been absorbed.

These proof-of-concept studies demonstrated that grape bunches of Chardonnay, Sauvignon Blanc and Semillon grapes, harvested at 19.8, 19.8 and 15.4 °Brix, respectively, were capable of forming a range of mono- and di-saccharides as a consequence of exposure to volatile phenols. The most abundant glycosides observed in the Chardonnay and Semillon grapes were the pentosylglucosides GuPG and CrPG and the monoglucosides GuMG and CrMG, as had been previously observed for table grapes. While these glycosides were also abundant in the Sauvignon Blanc grapes, an apparent variety effect was noted as Sauvignon Blanc grapes in addition contained elevated concentrations of the rutinosides of guaiacol, *o*-cresol and phenol (Table 2). The variation in glycoside concentrations between treatment 1 and treatment 2 reflects that repeat experiments were performed outdoors in separate desiccators with slightly different dimensions which may have resulted in variations in temperature conditions and evaporation rates between desiccators. Furthermore, differences may be attributable to variation in absorption of the volatile phenols and/or differences in enzymatic activity within the berries themselves. In summary, the results demonstrate that uptake and metabolism of volatile phenols by berries to the corresponding glycosides occur in excised bunches after they have been removed from the vine. In concurrent studies, similar effects were observed for excised grape bunches of Viognier and Cabernet Sauvignon that had been exposed to model smoke [31]. Consequently, the model system was considered suitable for screening a large number of horticultural products to identify potentially protective candidates prior to further investigations in field trials.

### 2.2. Evaluation of the Potential of Surface Coatings to Reduce Uptake of Smoke Taint Compounds by Grapes

Several experiments were conducted to investigate whether it is possible to limit the uptake of smoke volatile phenols by grapes through applying existing surface coatings that can be sprayed in the vineyard. To evaluate multiple products in parallel, the initial model system (desiccator with 6 L capacity) was upscaled to a larger 156 L glass container suitable for exposing multiple bunches in parallel to volatile phenols. Three separate experiments were performed, two with Muscat Gordo grapes and one with Shiraz grapes and each involved testing the twelve materials listed in Table 3. A more common white wine grape variety, such as Chardonnay, Sauvignon Blanc or Semillon, was not used as the grapes for these varieties were over-ripe and not suitable for use in these experiments by the time the model system had been developed and validated. Of the twelve surface coatings tested, nine are commercial products used in horticulture for reasons such as disease prevention, sunburn protection and anti-transpiration (Table 3). The other three materials were an activated carbon (FPS), titanium dioxide and silicone oil. The ability of activated carbon to adsorb organic compounds is quite well established [32] and it is known for its ability to scavenge organic compounds including volatile phenols in wine [33,34,35,36]. In this experiment activated carbon was included as a positive control, to see how much it might prevent uptake and glycosylation of volatile phenols by grapes. Titanium dioxide was chosen due to its ability to act as a catalyst in the breakdown of phenols in the presence of UV light activation [37]. Being solid materials, both carbon and titanium dioxide needed to be applied to grapes with support of a wetting agent. Therefore, they were dispersed in Raynox^®^ prior to application to grapes. While not necessarily a practical option as a spray in the vineyard, silicone oil was chosen due to its inert, oily and hydrophobic properties, and its inclusion could potentially provide further insights into adsorption and transport of volatile phenols.

**Figure 1:**
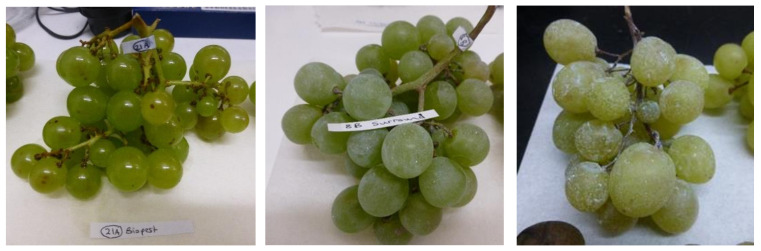
Muscat Gordo grape bunches after being treated with Biopest^®^ (**left**), Surround^®^WP (**middle**), and titanium dioxide in Raynox^®^ (**right**).

To achieve uniform coverage, the surface coatings were applied to grapes (Figure 1) by dipping, as opposed to spraying, the grape bunches into solutions or slurries of the horticultural products/materials in concentrations recommended by the manufacturer. The effectiveness of the treatments in preventing the uptake of volatile phenols by the grapes and their subsequent conversion to phenolic glycosides was evaluated by comparison to ‘non-treated’ control bunches (i.e., bunches not covered with any surface coatings). Both coated and control bunches were exposed to volatile phenols in the same glass container at the same time.

**Figure 2:**
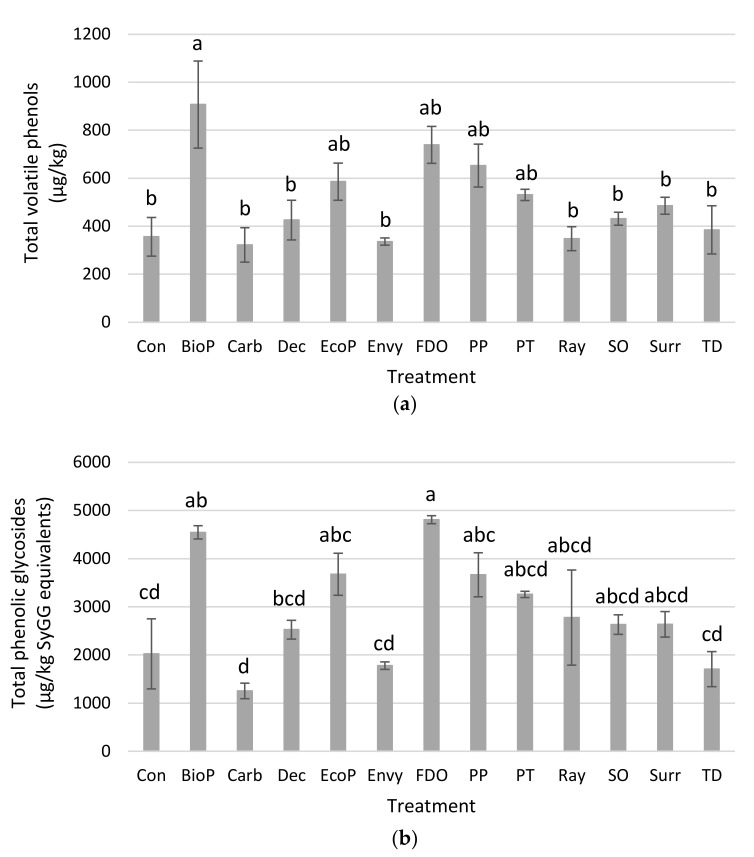
Concentrations of (**a**) total volatile phenols (n = 7) and (**b**) total phenolic glycosides (n = 15), contained in Muscat Gordo grape homogenates after grapes had been treated with surface coatings and then exposed to volatile phenols. Con = control; BioP = Biopest^®^; Carb = carbon (applied in Raynox^®^); Dec = Deccoshield; EcoP = Ecoprotector^®^; FDO = fruit drying oil; PP = Parka Plus; PT = Peratec; Ray = Raynox^®^; SO = silicone oil; Surr = Surround^®^WP; TD = titanium dioxide (applied in Raynox^®^). Different letters between treatments (annotated above the standard error bars) indicate statistical significance (*p* = 0.05, Tukey HSD means comparison test from one-way ANOVA).

**Figure 3:**
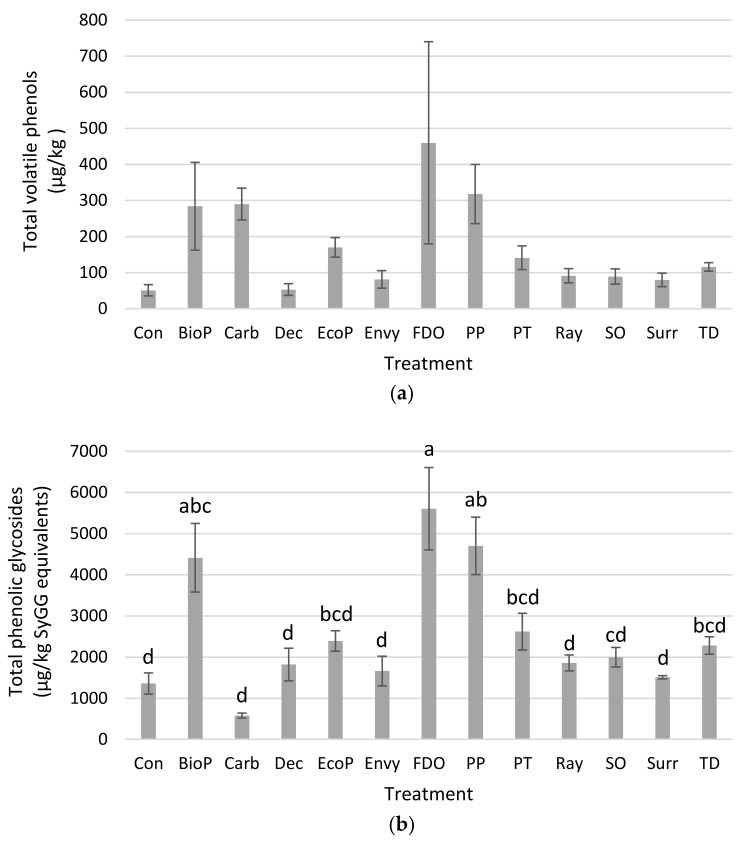
Concentrations of (**a**) total volatile phenols (n = 7) and (**b**) total phenolic glycosides (n = 15), contained in Shiraz grape homogenates after grapes had been treated with surface coatings and then exposed to volatile phenols. Con = control; BioP = Biopest^®^; Carb = carbon (applied in Raynox^®^); Dec = Deccoshield^®^; EcoP = Ecoprotector^®^; FDO = fruit drying oil; PP = Parka Plus; PT = Peratec; Ray = Raynox^®^; SO = silicone oil; Surr = Surround^®^WP; TD = titanium dioxide (applied in Raynox^®^). Different letters between treatments (annotated above the standard error bars) indicate statistical significance (*p* = 0.05, Tukey HSD means comparison test from one-way ANOVA).

The concentrations of total volatile phenols and total phenolic glycosides present in Muscat Gordo (Experiment 1) and Shiraz grape homogenates after grapes had been treated with surface coatings and then exposed to volatile phenols are presented graphically in Figure 2 and Figure 3, respectively. The results for each of the treatments for the repeat Muscat Gordo Experiment (Experiment 2) followed similar trends to that observed in the first experiment. Concentrations for the individual volatile phenols and phenolic glycosides for each of the three experiments are provided in Supplementary Tables S1–S6. The background concentrations of volatile phenols and phenolic glycosides contained in the starting Muscat Gordo and Shiraz grapes prior to exposure to volatile phenols (i.e., control—uncoated/no volatile phenol exposure) were very small and these values are also provided in Supplementary Tables S1–S6.

For the experiments on Muscat Gordo grapes, there was a trend towards increases in the volatile phenols contained in the grape homogenates when Biopest^®^, Ecoprotector^®^, Fruit Drying Oil, Parka Plus and Peratec were applied compared to the control (Figure 2a; Supplementary Tables S1 and S3). However, this increase was only found to be statistically significant for Biopest^®^. There were similar trends observed for the Shiraz grapes (Figure 3b; Table S5); however, none of the increases were found to be statistically significant most likely due to a large variability amongst replicates of the same treatment, particularly for the grape bunches treated with Biopest^®^, Fruit Drying Oil and Parka Plus. The variability after application by immersion into the protective materials is the likely consequence of differences in the size and quantities of grapes on individual bunches and potentially also in the grapes’ biochemical activity. It is likely to be even larger in field trials with spray-on application of the protective materials and products.

In this current study, none of the sunburn protectants (Deccoshield^®^, Raynox^®^, Surround^®^WP) was found to reduce the uptake of volatile phenols. In addition, the application of a cuticle supplement (i.e., Parka Plus) to the grape surface did also not provide any protection against the uptake of volatile phenols. The data are not consistent with the findings of a recent study which simulated exposure to forest fire smoke that suggested the application of an artificial grape cuticle one week prior to smoke exposure may significantly reduce volatile phenols at harvest [16]. It is unknown whether these differences relate to the timing and/or mode of the protective application, or conditions during smoke exposure, and further research is warranted to assess the effects of cuticular adjuncts.

The FPS activated carbon used in these experiments is highly effective at removing volatile phenols in smoke-effected grape juice but is relatively ineffective for phenolic glycosides [38]. The reduction in phenolic glycosides in both Muscat Gordo and Shiraz grapes demonstrated a certain protection the selected activated carbon might be able to provide when applied to grapes prior to smoke exposure. However, results from analyzing volatile phenols in grapes post exposure remained inconclusive, particularly for Shiraz, despite attempts with washing off carbons prior to homogenizing grapes to avoid interfering with the analytical methods. In addition, sunburn was observed for some carbon treated bunches, and complete coverage with spray-on carbon seemed elusive, hence the direct application of activated carbon to grapes was not further explored.

**Figure 4:**
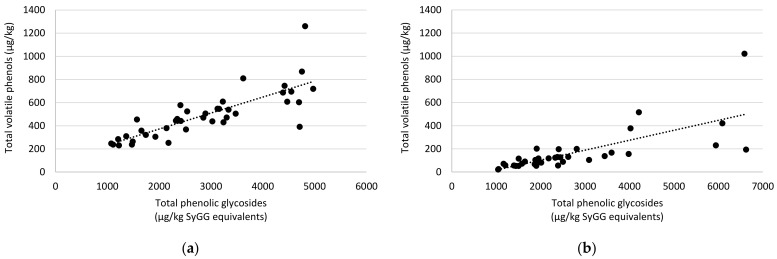
Concentrations of total volatile phenols plotted against total phenolic glycosides for (**a**) Muscat Gordo (Experiment 1) and (**b**) Shiraz grape homogenates after grapes had been treated with surface coatings and then exposed to volatile phenols. Note: Results for the carbon treatment were removed for Shiraz grapes since residual carbon interfered with the volatile phenol analysis.

When considering the concentrations of phenolic glycosides in the control and treated Muscat Gordo grapes (Figure 2b; Tables S2 and S4) and Shiraz grapes (Figure 3b; Table S6), good positive correlations were observed to the volatile phenol concentrations (Figure 2a and Figure 3a; Table S7). This is visualized when plotting the total volatile phenols against total phenolic glycosides for the Muscat Gordo and Shiraz Experiments (Figure 4). The correlations for concentrations of total volatile phenols against total glycosides had r values of 0.800, 0.688 and 0.728 for the Muscat Gordo (Experiment 1), Muscat Gordo (Experiment 2) and Shiraz Experiments, respectively. These positive correlations are expected since increased absorption of volatile phenols is likely to result in greater conversion to their glycosides. Consequently, as observed for the volatile phenol concentrations, the application of hydrophobic products (Biopest^®^, Fruit Drying oil and Parka Plus) resulted also in statistically significant increases in the total phenolic glycosides (and all individual glycosides measured) compared to those observed in the control (note: only significantly higher when using Parka Plus on Shiraz grapes). While for many treatments their application to the grape surface resulted in a trend towards increased mean concentrations of phenolic glycosides, they were not statistically higher than those contained in the control grapes. In some experiments the activated carbon and titanium dioxide coated grapes had somewhat lower average glycoside concentrations, but they were not significantly lower than the control. Any observed reductions for these grape coatings are likely attributable to the action of carbon and titanium dioxide and not the product they were applied in, since Raynox application alone has a trend towards increasing total glycoside concentrations (i.e., increase from 2024 to 2776, from 1207 to 2191 and from 1360 to 1858 µg/kg SyGG equivalents for Muscat Gordo Experiment 1, Muscat Gordo Experiment 2 and Shiraz, respectively), although none of these results were statistically higher than the control (Supplementary Tables S2, S4 and S6). Previous research by van der Hulst and colleagues (2019) found kaolin coated Merlot grapes exposed to smoke contained significantly lower phenolic glycoside concentrations than control (i.e., non-coated) grapes [14]. However, there was no significant effect of kaolin application on the phenolic glycosidic profiles of Sauvignon Blanc and Chardonnay grapes, and the same was observed in the current study (i.e., for Surround^®^WP). Discrepancies in results between grape varieties possibly may be attributed to the rate of application and extent of coverage [14], and further research is warranted.

As for the proof-of-concept experiments, higher concentrations of *o*-cresol and guaiacol were observed in the Muscat Gordo and Shiraz grape homogenates compared to *m*-cresol and syringol (Tables S1, S3 and S5), which, as mentioned previously, likely reflects the higher volatility of these phenols and hence the higher evaporation of them from the aqueous solution. Consequently, the glycosides of guaiacol and the cresols were the most abundant glycosides formed in the Muscat Gordo and Shiraz grapes (Tables S2, S4 and S6), with guaiacol gentiobioside (GuGG) and cresol glucoside (CrMG), consisting, on average, 45–50% of the total. The pentosylglucosides of guaiacol and the cresols comprised, on average, another 18–23% of the total glycosides, while cresol gentiobioside (CrGG) made up a further 7–10%. The glycosidic profiles were similar regardless of treatment. There was also some similarity in profiles between grape varieties, though guaiacol glucoside (GuMG) featured more prominently in Muscat Gordo grapes compared to Shiraz grapes (i.e., comprised an average of 12% of the total vs. 3%), while cresol rutinoside (CrRG) featured more prominently in Shiraz grapes compared to Muscat Gordo grapes (i.e., comprised an average of 14% of the total vs. 3%). These differences are likely to reflect variation in glycosyltransferases and/or the sugars between grape varieties.

**Figure 5:**
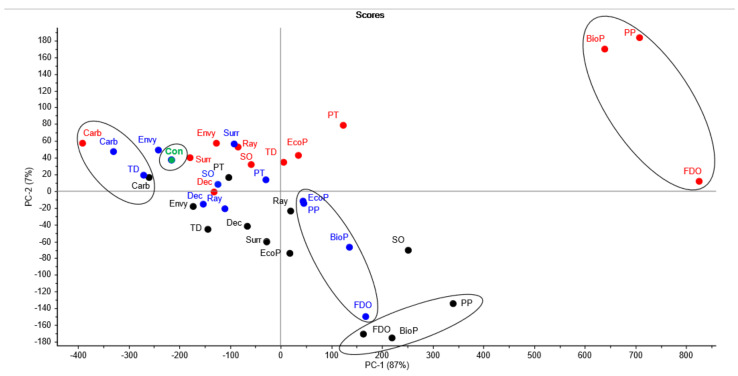
Principal component analysis for averaged individual phenolic glycosides data based on percentage to the control; black dot (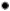) = Muscat Gordo Experiment 1; blue dot (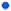) = Muscat Gordo Experiment 2; red dot (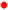) = Shiraz experiment; green dot (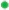) = Controls; Con = control; BioP = Biopest^®^; Carb = carbon; Dec = Deccoshield^®^; EcoP = Ecoprotector^®^; FDO = fruit drying oil; PP = Parka Plus; PT = peratec; Ray = Raynox^®^; SO = silicone oil; Surr = Surround^®^WP; TD = titanium dioxide.

To summarize and wholistically assess all results, principal component analysis was conducted on the most abundant individual phenolic glycosides (SyGG, GuGG, CrGG, GuPG, PhPG, CrPG, CrRG, GuMG, CrMG) for each treatment for each of the three experiments (i.e., triplicate data was averaged for 13 control/treatments × 3 experiments; n = 39) (Figure 5). The data was normalized across the three experiments by presenting the individual glycoside data for each of the treatments as a percentage to the control (Supplementary Tables S8–S10). This was required as the experiments were performed at different timepoints under varying ambient temperature ranges (i.e., 20.8–35.9 °C, 11.5–30.4 °C and 11.5–28.1 °C for Muscat Gordo Experiment 1, Muscat Gordo Experiment 2 and Shiraz, respectively). Therefore, not surprisingly, the concentrations of phenolic glycosides in the grapes were higher in the first Muscat Gordo Experiment compared to the second as the higher temperatures experienced in the first experiment encouraged greater volatilization of the volatile phenols from the aqueous volatile phenol solution. The concentrations of phenolic glycosides for many of the Shiraz treated grapes were similar to that observed for the second Muscat Gordo, with the exception being those coatings that seemed to encourage uptake of volatile phenols (i.e., Biopest^®^, Fruit Drying Oil, Parka Plus and Peratec) where concentrations were much higher. This outcome may be a consequence of the smaller berry sizes for Shiraz grapes compared to Muscat Gordo grapes, therefore resulting in a higher surface area to volume ratio.

PC-1 and PC-2 explained 87% and 7% of the variation in the data, respectively (Figure 5). Separation in PC-1 is based on treatment, while separation in PC-2 is based on grape variety (i.e., the majority of Muscat Gordo and all Shiraz treatments were located in the lower and upper quadrants, respectively). The controls reside in the upper-left quadrant and overlay for all three experiments (i.e., all represent a value of 100%). Any treatments situated to the left of the controls along the x-axis contained lower concentrations of total phenolic glycosides compared to the control and hence suggest that there was a reduction in the uptake of the volatile phenols in these grape bunches. In contrast, the treatments situated to the right of the controls along the x-axis are those that contain higher concentrations of total phenolic glycosides compared to the controls. The treatments located in the right quadrants, most notably, Biopest^®^, Fruit Drying Oil and Parka Plus, are least effective at mitigating the uptake of smoke compounds and this was consistent for all three experiments. For most treatments, there is good reproducibility between the two Muscat Gordo experiments with the same treatments being located in close proximity, though there are a few exceptions (i.e., Parka Plus, Peratec and silicone oil). Even the treatments between the Muscat Gordo and Shiraz grape varieties align relatively well on the y-axis, indicating similar trends were observed regardless of grape variety. When considering the loadings, separations were driven by the relative changes compared to the control for SyGG (0.625), GuMG (0.463), GuGG (0.425), CrGG (0.374) and CrMG (0.332) in PC-1 and SyGG (0.625), PhPG (−0.364), CrPG (−0.369) and GuPG (−0.458) in PC-2.

In summary, the absorption of volatile phenols by grapes was influenced by the surface coating applied, with none of the treatments tested providing effective protection against uptake. Higher concentrations of volatile phenols and their glycosidic metabolites were observed for grapes coated with Biopest^®^, Parka Plus and Fruit Drying Oil. The hydrophobic nature of these materials and/or the disruption to the grape cuticle by their application to the grape surface may be responsible for enhanced adsorption and/or transfer of volatile phenols into the berry.

### 2.3. The Importance of Efficient Grape Surface Coverage

While activated carbon and titanium dioxide may be capable of effectively reducing the levels of volatile phenols under certain conditions (for example, when added to grape musts or wine), the practicality of their use in the vineyard appears limited. Since both materials are solids, obtaining adequate coverage on grapes is an issue and would require large quantities to be used. Even with dipping grape bunches in slurries of carbon or titanium dioxide in Raynox (30 mg/mL), it was difficult to get good coverage of these materials on the grape surface. Furthermore, the use of these materials is not really feasible as they would need to be removed prior to grape processing and carbon, being black, acts as a heat sink and its presence on grapes can cause sunburn.

Good spray coverage in the vineyard is an important factor which must be considered if any coatings are found to prevent the update of volatile phenols into grape berries. Even for good disease control, it has been estimated that spray coverage of 80% or more is required [39]. For sunburn protection, spray coverage is not as crucial as those grape bunches located under the vine canopy have some protection from sunlight. However, the same is not true when smoke is present in the vineyard as the entire vine will be exposed to smoke. In one study, the variability in efficacy of kaolin treatment for grapes prior to smoke exposure was attributed to the rate of application and extent of coverage [14], further highlighting the difficulty in obtaining good spray coverage for solid materials such as clay.

## 3. Materials and Methods

### 3.1. Chemicals, Horticultural Products and Materials

Solvents (HPLC grade) were purchased from Merck (Darmstadt, Germany). Deuterium-labelled internal standards (i.e., *d*_3_-guaiacol, *d*_3_-4-methylguaiacol, *d*_7_-*o*-cresol, *d*_3_-syringol, *d*_3_-guaiacol *β*-D-glucoside and d_3_-syringol gentiobioside) were synthesized in-house, as previously reported [2,5,40].

The horticultural products and materials were sourced from the following suppliers: Biopest^®^ Paraffinic Oil from SACOA Pty Ltd. (Claremont, WA, Australia); FPS activated carbon from Vason Group (Verona, Italy); Deccoshield^®^ from Decco Italia s.r.l. (Piano Tavola, Catania, Italy); Ecoprotector^®^ from Organic Crop Protectants Pty Ltd. (Lilyfield, NSW, Australia); Envy from AgroBest Australia Pty Ltd. (Nerang, QLD, Australia); Victoria Fruit drying oil (sold in Australia as Vouillaire’s Eemulsoyle) from Victorian Chemical Co. Pty Ltd. (Coolaroo, VIC, Australia); Parka Plus from InSense Pty Ltd. (Cobram, VIC, Australia); Peratec Plus from Jaegar Australia (Noble Park, VIC, Australia); Raynox from Collin Campbell (Chemicals) Pty Ltd. (Wetherill Park, NSW, Australia); Silicone oil 47v50 to 47v1000 from Victoria Lub Pty Ltd. (Keysborough, VIC, Australia); Surround^®^WP from AgNova Technologies Pty Ltd. (Box Hill North, VIC, Australia); and Titanium dioxide from Sigma Aldrich Inc. (Castle Hill, NSW, Australia).

### 3.2. Table and Wine Grapes

Table grapes (unknown variety, country of origin, harvest date and storage time) were purchased from local supermarkets in South Australia. Chardonnay, Sauvignon Blanc and Semillon grapes (600–700 g for each) were harvested at 19.8, 19.8 and 15.4 °Brix, respectively, in February 2017 from the Coombe Vineyard at the University of Adelaide’s Waite Campus in Urrbrae, South Australia (34°58′ S, 138°38′ E). Grapevines were planted (in 1998) in north–south aligned rows on their own roots; trained to a bilateral cordon, vertical shoot positioned trellis system; hand-pruned to a two-node spur system; and drip irrigated. Muscat Gordo grapes (approx. 20 kg) and Shiraz grapes (approx. 10 kg) were harvested from the SARDI Research Centre (Nuriootpa, South Australia) in February 2017. Grape bunches were stored at 5 °C until needed.

### 3.3. Absorption of Gaseous Phenols by Excised Grape Bunches—Proof-of-Concept Experiments

#### 3.3.1. Preparation of Volatile Phenol Mixture (300 mg/L)

Guaiacol (0.12 g), *m*-cresol (0.12 g), *o*-cresol (0.12 g), 2,6-dimethoxyphenol (0.12 g) and phenol (0.12 g) were weighed into a volumetric flask (200 mL). The flask was made up to the mark with deionized water making a solution with a concentration of 600 mg/L of each of the phenols (or collectively 3 g/L). This solution was further diluted 1 in 10 to give a concentration of 60 mg/L of each of the phenols (or collectively 300 mg/L).

#### 3.3.2. Exposure of Table Grapes and Wine Grapes to Gaseous Volatile Phenols

**Table grapes:** Initial experiments were performed with table grapes (variety unknown) due to their availability outside of vintage. Grape bunches (n = 2; 150–250 g each) were placed into a glass desiccator (volume of approximately 6 L) which contained an aqueous volatile phenols mix (300 mg/L, 20 mL) in a glass Pyrex dish. A second desiccator contained control grape bunches (n = 2; 150–210 g each) which were not exposed to volatile phenols (i.e., contained 20 mL of deionized water in a glass Pyrex dish). Desiccators were left outside (unstoppered) for approximately 60 h (2–5 December 2016). After this time, grape bunches were placed in individual zip lock plastic bags and frozen at −20 °C until ready for volatile phenols and phenolic glycoside analysis. **Wine grapes:** One bunch (128–142 g) of each of the white wine grape varieties (Chardonnay, Sauvignon Blanc and Semillon) was placed in two desiccators which contained an aqueous volatile phenols mix (300 mg/L, 20 mL) in a glass Pyrex dish. Both desiccators were left outside (unstoppered) for approximately 60 h (14–17 February 2017), where maximum daily temperatures were 22–30 °C and minimum night temperatures were 15–20 °C. After this time, grape bunches were placed in individual zip lock plastic bags and frozen at −20 °C until ready for volatile phenols and phenolic glycoside analysis.

### 3.4. Evaluation of Horticultural Products to Reduce Uptake of Smoke Taint Compounds by Grapes

#### 3.4.1. Preparation of Horticultural Products and Materials

Solutions of the horticultural products/materials were prepared in concentrations recommended by the manufacturer via dilution in MilliQ water and were as follows. Biopest^®^: 15 mL added to water (485 mL); Carbon: 30 mg/mL solution in Raynox^®^ (1 in 40 dilution); Deccoshield^®^: 10 mL added to water (490 mL); Ecoprotector^®^: 10 mL added to water (490 mL); Envy: 25 mL added to water (475 mL); Victoria fruit drying oil: 7.5 mL added to water (492.5 mL) containing 10 g potassium carbonate; Parka Plus: 5 mL added to water (495 mL); Peratec: 5 mL added to water (495 mL); Raynox^®^: 10 mL added to water (390 mL); Silicone oil: used “as is”, no preparation required; Surround: 25 g added to water (500 mL) plus the addition of wetter (Viti-Wet (SST Australia Pty Ltd., Bayswater, Victoria); 25 µL); Titanium dioxide: 30 mg/mL in Raynox^®^ (1 in 40 dilution).

#### 3.4.2. Model System for Exposure of Control and Treated Excised Wine Grape Bunches to Gaseous Volatile Phenols

Three separate experiments were performed, two on Muscat Gordo grapes and one on Shiraz grapes, testing twelve different surface coatings (Table 2) alongside controls consisting of uncoated grape bunches. Treatments (including the uncoated controls) were applied in triplicate, with the aim of having grape bunches weigh typically 150–250 g. Grapes were coated by dipping in the prepared solutions with the materials to be tested and left to dry on paper towel after dipping (Figure 1).

For each experiment, all coated grape berries and the uncoated controls were placed inside a glass vessel (commercially purchased fish tank, external dimensions 910 mm × 380 mm × 450 mm, 156 L capacity) at the same time and exposed to gaseous volatile phenols. Within the base of the tank, 6 × Pyrex dishes (11.5 cm in diameter, 470 mL capacity) were evenly placed. Aqueous phenol solution (total phenols 1.3 g/L, 20 mL) was placed in each of the Pyrex dishes and two dishes were under each of three custom made stainless steel platforms (29.5 cm × 36.5 cm × 11.5 cm), each containing 17 × 21 holes (5 mm in diameter).

The aqueous phenol solution consisted of guaiacol, *m*-cresol, *o*-cresol, 2,6-dimethoxyphenol (syringol) and phenol. It was prepared by dissolving 120 mg of each of the phenols in 200 mL of MilliQ water (i.e., 600 mg/L of each phenol, collectively 3 g/L). This stock solution was diluted 1 in 2.3 with MilliQ water to give a solution containing 260 mg/L of each phenol. With 120 mL of this solution used in the experiments, 20 mL in each of six Pyrex dishes, approximately 31.2 mg of each phenol was contained in that 120 mL quantity, or collectively 156 mg, in a tank of 156 L capacity. Grape bunches were randomly placed on the stainless-steel platforms, arranged to ensure there was one of each treatment on each of the three platforms. A data logger recording temperature and humidity was placed inside the glass vessel. The glass lids were placed on the top of the fish tank and a small fan was used to circulate air within the tank. The grapes remained in the vessel for 60 h. Specific details for each of the three experiments were as follows. **Muscat Gordo Experiment 1**: Experiment started at 9 pm on 24 March 2017 and finished at 9 am on 27 March 2017. Range of bunch weights: first replicate 205–232 g; second replicate: 157–175 g; third replicate: 112–165 g. For the duration of the experiment, the temperature in the tank ranged from 20.8 °C to 35.9 °C. **Muscat Gordo Experiment 2**: Experiment started at 9 pm on 4 April 2017 and finished at 9 am on 7 April 2017. Range of bunch weights: first replicate 194–255 g; second replicate: 150–188 g; third replicate: 132–157 g. For the duration of the experiment, the temperature in the tank ranged from 11.5 °C to 30.4 °C. **Shiraz Experiment**: Experiment started at 9 pm on 23 April 2017 and finished at 9 am on 26 April 2017. Range of bunch weights: first replicate 193–237 g; second replicate: 145–183 g; third replicate: 122–152 g. For the duration of the experiment, the temperature in the tank ranged from 11.5 °C to 28.1 °C.

After being exposed to gaseous phenols in a fish tank for 60 h, grape bunches underwent a semi-abrasive cleaning procedure to remove as much of the coating as possible (particularly important for carbon coated grapes as the presence of carbon in the grape homogenate may interfere with the analysis). It was difficult to remove the oily substances, particularly difficult for silicone oil. Tissues and wipes were used to wipe off as much oil as possible but there was still some residue. Grape bunches were then placed in individual zip lock plastic bags and stored at −20 °C until ready for volatile phenols and phenolic glycoside analysis.

### 3.5. Chemical Analysis for Smoke Taint Compounds

#### 3.5.1. Preparation of Grape Homogenates

When ready for homogenization and extraction, grapes were defrosted at ambient temperature, all berries were removed from stalks and the number of berries counted and weighed. All berries were homogenized at 8000 rpm for 20 s using a Grindomix GM200 blender (Retsch GmbH, Haan, Germany). Homogenate samples, 25 g and 5 g, were weighed out in 50 and 10 mL centrifuge tubes for volatile phenol and phenolic glycosides analysis, respectively.

#### 3.5.2. Volatile Phenol Analysis

The guaiacol, 4-methylguaiacol, *m*-, *o*- and *p*-cresols, syringol and methylsyringol concentration in grape samples was quantitated, against calibration curves consisting of each of those individual volatile phenols and using gas chromatography mass spectrometry (GCMS) and stable isotope dilution methods reported previously [2,40,41]. Internal standards, d_3_-guaiacol (for guaiacol), d_3_-4-methylguaiacol (for 4-methylguaiacol), d_3_-syringol (for *o*-cresol, *m*-cresol, syringol and 4-methylsyringol) and d_7_-*p*-cresol (for *p*-cresol) were synthesized via methods previously described [40]. Analysis was performed by the AWRI’s Commercial Services Laboratory.

#### 3.5.3. Phenolic Glycoside Analysis

Smoke glycosides were quantified using stable isotope dilution analysis and liquid chromatography mass spectrometry (LCMS) with d_3_-syringol gentiobioside (d_3_-SyGG) used as the internal standard [2]. Grape homogenate (5 g) was spiked with d_3_-SyGG (25 µL of 100 µg/mL solution) to give a final concentration in the homogenate of 500 μg/kg. After mixing by vortex, samples were centrifuged (3500 rpm for 5 min) and a portion of juice supernatant (2 mL) was extracted by solid phase extraction according to the method used by Hayasaka et al., 2013 [5].

### 3.6. Statistical Analysis

Chemical data were analyzed using a combination of descriptive and multivariate techniques, including one-way ANOVA with post hoc Tukey’s test (*p* = 0.05), correlation analysis (Pearson, 95% confidence level) and principal component analysis (PCA) using Minitab 18 (Minitab Inc., Sydney, NSW) and The Unscrambler (version 11, Camo Analytics, Oslo, Norway), respectively.

## 4. Conclusions

Excised table and wine grape bunches are capable of forming a range of mono- and di-saccharides as a consequence of exposure to volatile phenols, with the glycoside profiles broadly similar to what has been previously observed after smoke exposure of grapes on vines. A model system was developed that was capable of evaluating the effectiveness of applying a broad range of horticultural product surface coatings to grapes for reducing/preventing the uptake of volatile phenols guaiacol, syringol, *o*-cresol, *m*-cresol and phenol. This model system was used to assess potentially protective candidates prior to undertaking further investigations in field trials. None of the treatments tested were found to provide effective protection of grapes from volatile phenols. It is important to note that the commercial horticultural products evaluated in this study are not marketed to reduce smoke taint but are recommended for other preventative measures such as pest and disease management and sunburn protection. The highest concentrations of volatile phenols and their glycosidic grape metabolites (i.e., increases compared to untreated control grapes) were observed for the more oily, hydrophobic materials; this indicates that the hydrophobic nature of these materials may enhance the adsorption and/or transfer of volatile phenols from smoke into grape berries. Therefore, it is important to consider what sprays are being applied in the vineyard before and during smoke events to prevent unintentionally exacerbating the uptake of smoke compounds by grapes.

## Figures and Tables

**Table 1:** Concentrations of volatile phenols and corresponding phenolic glycosides in table grapes after exposure to gaseous volatile phenols (treated) or without exposure (control).

Concentration ^1^ (µg/kg)												
Sample	Gu	GuGG	GuPG	GuRG	GuMG	*o*-Cr	*m*-Cr	CrPG	CrRG	CrMG	Sy	SyGG
control 1	<1	<1	1	<1	<1	<1	<1	<1	<1	<1	<2	<1
control 2	<1	<1	1	<1	<1	<1	<1	1	<1	<1	<2	<1
treated 1	114	40	324	19	456	211	52	321	39	562	3	10
treated 2	192	69	410	24	506	304	85	463	54	730	8	27

^1^ GuGG, GuPG, GuRG, GuMG, CrPG, CrRG, CrMG and SyGG are measured as syringol glucose-glucoside equivalents; Gu = guaiacol; Cr = cresol; Sy = syringol; GG = glucose-glucoside (gentiobioside); PG = pentose-glucoside; RG = rutinoside; MG = monoglucoside.

**Table 2:** Concentrations of phenolic glycosides in Chardonnay, Sauvignon Blanc and Semillon grapes after exposure to gaseous volatile phenols (treated) or without exposure (control).

	Concentrations (µg/kg SyGG Equivalents)										
Sample	GuGG	GuPG	GuRG	GuMG	CrPG	CrRG	CrMG	SyGG	PhPG	PhRG	Total ^1^
Chardonnay Grapes											
control 1	<1	5	<1	<1	9	<1	4	<1	12	<1	30
treated 1	88	1882	61	302	2953	232	1055	31	263	8	6875
treated 2	11	502	16	90	960	58	414	2	84	3	2140
Sauvignon Blanc grapes											
control 1	<1	2	<1	<1	6	2	3	<1	6	<1	19
treated 1	134	410	374	543	1012	894	1621	11	66	70	5135
treated 2	36	81	120	115	176	213	382	<1	21	18	1162
Semillon grapes											
control 1	<1	3	<1	<1	8	<1	3	<1	7	<1	21
treated 1	21	985	77	609	2153	292	1330	4	264	18	5753
treated 2	16	626	59	269	1445	203	1004	2	136	12	3772

^1^ Total = the sum for the ten glycosides listed in the table. Gu = guaiacol; Cr = cresol; Sy = syringol; Ph = phenol; GG = glucose-glucoside (gentiobioside); PG = pentose-glucoside; RG = rutinoside; MG = monoglucoside.

**Table 3:** Horticultural product surface coatings investigated for their ability to prevent uptake of gaseous phenols by wine-grapes and their typical application.

Product	Typical Application
Biopest^®^	Pest management
FPS Carbon	Chemical scavenger
Deccoshield^®^	Sunburn protectant
Ecoprotector^®^	Pest management
Envy	Anti-transpirant
Victorian fruit drying oil	Vine fruit drying
Parka Plus	Cuticle supplement
Peratec	Pest management
Raynox^®^	Sunburn protectant
Silicone oil	Lubricant
Surround^®^WP	Sunburn protectant
Titanium dioxide	Color additive

## Data Availability

The data presented in this study are available in Supplementary Material.

## Supplementary Materials

Table S1: Concentrations of volatile phenols (µg/kg) in Muscat Gordo grape homogenates after grapes were covered with various surface coatings and exposed to gaseous volatile phenols for 60 h (Experiment 1); Table S2: Concentrations of phenolic glycosides (µg/kg) in Muscat Gordo grape homogenates after grapes were covered with various surface coatings and exposed to gaseous volatile phenols for 60 h (Experiment 1); Table S3: Concentrations of volatile phenols (µg/kg) in Muscat Gordo grape homogenates after grapes were covered with various surface coatings and exposed to gaseous volatile phenols for 60 h (Experiment 2); Table S4: Concentrations of phenolic glycosides (µg/kg) in Muscat Gordo grape homogenates after grapes were covered with various surface coatings and exposed to gaseous volatile phenols for 60 h (Experiment 2); Table S5: Concentrations of volatile phenols (µg/kg) in Shiraz grape homogenates after grapes were covered with various surface coatings and exposed to gaseous volatile phenols for 60 h; Table S6: Concentrations of phenolic glycosides (µg/kg) in Shiraz grape homogenates after grapes were covered with various surface coatings and exposed to gaseous volatile phenols for 60 h; Table S7: Correlations between the concentrations guaiacol, o-cresol, p-cresol, total volatile phenols and various aglycone groups of glycosides and total glycosides contained in Muscat Gordo and Shiraz grape homogenates after grapes were covered in various surface coatings and exposed to volatile phenols for 60 h; Table S8: Percentage compared to the control for the most abundant phenolic glycosides in Muscat Gordo grape homogenates after grapes were covered with various surface coatings and exposed to gaseous volatile phenols for 60 h (Experiment 1); Table S9: Percentage compared to the control for the most abundant phenolic glycosides in Muscat Gordo grape homogenates after grapes were covered with various surface coatings and exposed to gaseous volatile phenols for 60 h (Experiment 2); Table S10: Percentage compared to the control for the most abundant phenolic glycosides in Shiraz grape homogenates after grapes were covered with various surface coatings and exposed to gaseous volatile phenols for 60 h.

## Sample Availability

Samples of the compounds are not available from the authors.
