# Supplementary material for: Development and Utilization of a Model System to Evaluate the Potential of Surface Coatings for Protecting Grapes from Volatile Phenols Implicated in Smoke Taint
Source: Molecules. 2021 Aug 27;26(17):5197. doi: 10.3390/molecules26175197 (PMC8434455; doi:10.3390/molecules26175197)
Supplement: Supplementary file 1 [file molecules-26-05197-s001.zip › molecules-1309541-supplementary.pdf]

Supplementary Materials

Development and utilization of a model system to evaluate the potential of surface coatings for protecting grapes from volatile phenols implicated in smoke taint

Julie A. Culbert [1,*], Mark P. Krstic [1,] and Markus J. Herderich [1]

[1] The Australian Wine Research Institute, PO Box 197, Glen Osmond, SA, 5064, Australia julie.culbert@awri.com.au (J.C.); mark.krstic@awri.com.au (M.K.); markus.herderich@awri.com.au (M.H.);

* Correspondence: julie.culbert@awri.com.au (J.C.); Tel.: +61-8-8313-6600

Table S1. Concentrations of volatile phenols (μg/kg) in Muscat Gordo grape homogenates after grapes were covered with various surface coatings and exposed to gaseous volatile phenols for 60 hours (experiment 1)

	Concentration (μg/kg)				
**Treatment**	**Guaiacol**	***o*-Cresol**	***m*-Cresol**	**Syringol**	**Total**
Control (uncoated/no VP exposure)	<1	<1	<1	<2	<2
Control (uncoated/VP exposed)	80 ± 16.6 b	219 ± 51.0 c	49 ± 14.0 b	<2	348 ± 81.4 b
Biopest®	196 ± 51.7 a	569 ± 98.2 a	133 ± 32.8 a	<2	898 ± 182.1 a
Carbon[a]	72 ± 15.2 b	202 ± 44.2 c	40 ± 11.5 b	<2	314 ± 70.9 b
Deccoshield®	87 ± 17.0 b	270 ± 53.1 bc	60 ± 13.8 ab	<2	417 ± 83.1 b
Ecoprotector®	115 ± 21.2 ab	374 ± 41.2 abc	89 ± 16.3 ab	2.3 ± 1.2	580 ± 78.3 ab
Envy	71 ± 7.8 b	211 ± 7.0 c	46 ± 2.9 b	<2	328 ± 15.5 b
Fruit drying oil	154 ± 14.5 ab	473 ± 50.3 ab	104 ± 12.5 ab	<2	731 ± 76.7 ab
Parka Plus	136 ± 25.6 ab	407 ± 44.6 abc	98 ± 19.1 ab	3.7 ± 2.7	645 ± 89.3 ab
Peratec	109 ± 7.7 ab	333 ± 12.7 abc	78 ± 3.8 ab	3 ± 1.7	523 ± 24.0 ab
Raynox®	73 ± 8.4 b	220 ± 33.4 c	44 ± 7.8 b	<2	337 ± 49.3 b
Silicone oil	88 ± 5.9 b	274 ± 16.1 bc	60 a± 5.5 b	<2	422 ± 27.5 b
Surround®WP	100 ± 2.1 ab	301 ± 25.8 bc	76 ± 7.0 ab	<2	477 ± 34.3 ab
Titanium dioxide[a]	85 ± 25.5 b	240 ± 61.3 bc	51 ± 14.7 b	<2	376 ± 101.3 b
P	0.006	<0.001	0.002	ns	<0.001

Values are means ± standard error of three replicates (n = 3); Different letters within columns indicate statistically significant differences between treatment means (*P* = 0.05, one-way ANOVA); ns = not significant; Control (uncoated/no VP exposure) is not included in the statistical analysis and values listed are for a single replicate; MSy = 4-methylsyringol. [a]Applied in Raynox®; VP = volatile phenol.

Table S2. Concentrations of phenolic glycosides (µg/kg) in Muscat Gordo grape homogenates after grapes were covered with various surface coatings and exposed to gaseous volatile phenols for 60 hours (experiment 1)

		Concentration (µg/kg SyGG equivalents)															
**Treatment**		**SyGG**	**GuGG**	**PhGG**	**CrGG**	**SyPG**	**GuPG**	**PhPG**	**CrPG**	**GuRG**	**PhRG**	**CrRG**	**SyMG**	**GuMG**	**PhMG**	**CrMG**	**Total**
Control (uncoated/ no VP exposure)		1	<1	<1	4	<1	1	3	3	<1	<1	1	<1	2	<1	<1	15
Control (uncoated/ VP exposed)	Average	88 abc	321 bc	4.0 abc	123 d	2.8 ab	197 abc	38 ab	299 ab	10.3 abc	10.2 ab	67 bcd	3.9 ab	298 b	4.4	558 ab	2024 cd
	Std Error	37.1	133.8	1.7	44.9	0.8	54.4	11.9	91.3	4.0	3.8	22.7	1.6	106.2	1.1	228.3	728.5
Biopest®	Average	151 ab	937 a	6.7 abc	375 ab	3.9 ab	347 ab	46 ab	453 ab	22.9 a	16.0 ab	149 ab	5.2 ab	723 a	14.1	1294 a	4544 ab
	Std Error	18.8	71.7	0.4	16.5	0.5	29.8	6.0	60.6	0.5	2.5	16.1	0.7	50.1	8.0	74.8	138.0
Carbon[a]	Average	23.5 c	216 c	1.1 c	87 d	1.0 b	138 c	28 b	213 b	4.8 c	6.9 b	33 d	1.0 b	174 b	2.0	325 b	1254 d
	Std Error	4.7	37.1	0.2	23.6	0.1	15.2	0.4	33.7	0.7	1.6	7.8	0.2	32.9	0.6	63.1	159.9
Deccoshield®	Average	110 abc	367 bc	5.7 abc	159 cd	3.3 ab	246 abc	54 ab	466 ab	10.1 abc	13.4 ab	94 bcd	3.9 ab	295 b	2.7	695 ab	2525 cd
	Std Error	10.7	38.7	0.6	16.6	0.3	21.1	8.0	68.2	1.1	1.4	6.2	0.8	66.9	0.4	147.5	193.9
Ecoprotector®	Average	199 a	729 ab	10.0 a	304 abc	5.9 a	288 abc	57 ab	473 ab	13.9 abc	17.2 ab	131 abc	6.7 ab	424 ab	5.8	1010 ab	3675 abc
	Std Error	44.2	26.8	3.0	36.2	0.8	63.7	13.5	125.1	2.8	1.3	8.5	1.2	67.1	1.0	90.7	438.3
Envy	Average	89 abc	328 bc	4.3 abc	121 d	2.6 ab	170 bc	37 ab	270 b	6.1 bc	8.4 ab	64 cd	3.1 ab	187 b	2.2	485 b	1778 cd
	Std Error	9.2	45.9	0.5	4.0	0.4	20.9	2.0	42.6	1.4	0.7	6.5	0.5	30.9	0.3	60.2	79.3
Fruit drying oil	Average	149 ab	1035 a	7.2 abc	426 a	3.7 ab	385 a	71 a	655 a	20.4 ab	21.8 a	181 a	4.0 ab	576 ab	7.2	1267 a	4809 a
	Std Error	20.0	56.6	0.8	17.6	0.3	38.2	13.6	75.4	4.4	2.7	9.8	0.5	99.8	0.7	75.0	83.2
Parka Plus	Average	173 a	688 ab	8.0 ab	363 ab	4.7 ab	267 abc	42 ab	456 ab	18.3 abc	15.1 ab	123 abc	4.5 ab	497 ab	6.3	999 ab	3665 abc
	Std Error	27.7	55.9	1.7	38.2	1.0	56.0	8.2	110.0	1.1	1.4	12.3	0.7	73.3	1.5	109.6	455.7
Peratec	Average	195 a	359 bc	8.8 a	205 bcd	4.5 ab	294 abc	57 ab	429 ab	20.7 ab	17.8 ab	124 abc	7.6 a	513 ab	10.9	1012 ab	3258 abcd
	Std Error	4.6	111.9	0.2	14.4	0.2	10.6	10.3	18.8	4.0	4.2	20.3	0.1	24.8	7.2	44.2	65.8
Raynox®	Average	103 abc	454 bc	4.7 abc	219 bcd	4.4 ab	258 abc	44 ab	431 ab	15.0 abc	14.2 ab	100 abcd	5.1 ab	397 ab	2.9	724 ab	2776 bcd
	Std Error	26.8	170.9	0.9	85.2	2.1	41.7	7.5	68.7	6.5	5.9	37.8	2.8	189.8	0.4	354.6	986.9
Silicone oil	Average	124 abc	399 bc	6.0 abc	168 cd	3.2 ab	233 abc	52 ab	380 ab	14.6 abc	14.2 ab	99 abcd	4.7 ab	407 ab	2.4	725 ab	2632 bcd
	Std Error	9.3	22.8	0.6	9.9	0.1	32.2	4.9	52.1	1.4	1.3	1.1	0.6	32.3	0.3	90.4	203.6
Surround®WP	Average	168 a	484 bc	8.4 ab	224 bcd	4.2 ab	189 bc	40 ab	320 ab	12.5 abc	12.9 ab	99 abcd	5.6 ab	331 ab	8.7	729 ab	2636 bcd
	Std Error	29.7	75.7	1.6	34.1	0.6	7.8	2.0	7.0	1.8	1.2	13.7	1.2	35.8	4.5	90.9	263.4
TiO2[a]	Average	49 bc	237 c	2.3 bc	110 d	1.7 b	196 abc	34 ab	305 ab	7.9 abc	9.1 ab	59 cd	2.1 ab	231 b	6.4	453 b	1704 cd
	Std Error	6.9	42.5	0.4	20.0	0.2	46.3	5.7	69.8	1.8	1.4	11.6	0.5	53.5	4.7	117.1	362.5
	P	<0.001	<0.001	0.001	<0.001	0.013	0.004	ns	0.019	0.002	0.028	<0.001	0.024	0.001	ns	0.001	<0.001

Values are means and standard errors of three replicates (n = 3) measured as syringol glucose-glucoside equivalents; Different letters within columns indicate statistically significant differences between treatment means (P = 0.05, one-way ANOVA); ns = not significant; Control (uncoated/no VP exposure) is not included in the statistical analysis and values listed are for a single replicate; Sy = syringol; Gu = guaiacol; Ph = phenol; Cr = cresol; GG = glucose-glucoside (gentiobioside); PG = pentose-glucoside; RG = rutinoside; MG = monoglucoside. [a]Applied in Raynox®; VP = volatile phenol.

Table S3. Concentrations of volatile phenols (µg/kg) in Muscat Gordo grape homogenates after grapes were covered with various surface coatings and exposed to gaseous volatile phenols for 60 hours (experiment 2)

	Concentration (µg/kg)				
**Treatment**	**Guaiacol**	***o*-Cresol**	***m*-Cresol**	**Syringol**	**Total**
Control (uncoated/no VP exposure)	<1	<1	<1	<2	<2
Control (uncoated/VP exposed)	67 ± 7.9 b	188 ± 23.9 b	42 ± 6.4 c	<2	297 ± 38.2 c
Biopest®	143 ± 21.5 ab	385 ± 49.4 ab	86 ± 12.8 abc	<2	614 ± 81.6 abc
Carbon[a]	99 ± 32.6 b	204 ± 51.1 b	48 ± 14.4 c	<2	351 ± 98.0 c
Deccoshield®	59 ± 4.9 b	189 ± 9.3 b	40 ± 2.6 c	<2	288 ± 16.5 c
Ecoprotector®	91 ± 25.8 b	271 ± 46.9 b	60 ± 11.7 c	<2	422 ± 83.7 bc
Envy	59 ± 7.8 b	154 ± 19.3 b	35 ± 4.5 c	<2	248 ± 31.1 c
Fruit drying oil	319 ± 119.4 a	597 ± 116.0 a	150 ± 32.2 a	<2	1066 ± 267.2 a
Parka Plus	230 ± 24.4 ab	538 ± 70.0 a	131 ± 26.2 ab	2 ± 1.0	901 ± 120.0 ab
Peratec	81 ± 3.3 b	246 ± 13.1 b	59 ± 4.7 c	<2	386 ± 20.5 c
Raynox®	79 ± 9.8 b	234 ± 18.8 b	55 ± 4.4 c	<2	368 ± 32.4 c
Silicone oil	93 ± 12.5 b	269 ± 25.0 b	63 ± 3.5 bc	<2	425 ± 40.9 bc
Surround®WP	72 ± 14.0 b	198 ± 20.9 b	43 ± 4.0 c	<2	313 ± 37.3 c
Titanium dioxide[a]	71 ± 5.2 b	210 ± 19.0 b	46 ± 3.7 c	<2	327 ± 27.5 c
P	0.001	<0.001	<0.001	ns	<0.001

Values are means ± standard error of three replicates (n = 3); Different letters within columns indicate statistically significant differences between treatment means ($P = 0.05$, one-way ANOVA); ns = not significant; Control (uncoated/no VP exposure) is not included in the statistical analysis and values listed are for a single replicate; MSy = 4-methylsyringol. [a]Applied in Raynox®; VP = volatile phenol.

Table S4. Concentrations of phenolic glycosides (μg/kg) in Muscat Gordo grape homogenates after grapes were covered with various surface coatings and exposed to gaseous volatile phenols for 60 hours (experiment 2)

		**Concentration (μg/kg SyGG equivalents)**															
**Treatment**		**SyGG**	**GuGG**	**PhGG**	**CrGG**	**SyPG**	**GuPG**	**PhPG**	**CrPG**	**GuRG**	**PhRG**	**CrRG**	**SyMG**	**GuMG**	**PhMG**	**CrMG**	**Total**
Control (uncoated/ no VP exposure)		1	<1	<1	6	<1	1	2	2	<1	<1	2	<1	3	<1	<1	17
Control (uncoated/	Average	52 ab	305 bc	2.5 ab	114 b	2.1 c	78 b	18.1	124 bc	6.7 a	7.8	48 bc	1.1 ab	118 c	2.8	327 b	1207 bc
VP exposed)	Std Error	12.4	18.1	0.5	7.0	0.4	2.4	1.0	9.6	0.7	0.9	4.7	0.3	18.7	0.8	44.2	86.0
Biopest®	Average	92 ab	729 abc	4.4 ab	312 ab	3.1 abc	246 a	33.6	326 ab	13.5 a	10.9	94 ab	2.3 ab	430 a	4.4	914 ab	3215 ab
	Std Error	15.6	31.4	0.9	10.9	0.5	23.4	4.9	40.2	1.4	1.3	6.3	0.4	17.0	1.6	89.1	148.1
Carbon[a]	Average	20 b	230 c	1.1 b	106 b	1.2 c	74 b	18.8	103 c	5.8 a	7.4	32 c	0.5 b	141 c	2.3	326 b	1069 c
	Std Error	6.0	63.3	0.3	30.1	0.4	5.6	1.9	2.3	2.3	2.4	8.8	0.1	34.1	1.0	54.7	211.7
Deccoshield®	Average	85 ab	399 abc	4.2 ab	190 ab	2.7 bc	138 ab	30.8	231 abc	7.6 a	9.1	63 abc	1.5 ab	176 c	2.9	496 ab	1837 abc
	Std Error	6.7	28.3	0.4	21.7	0.4	12.9	3.3	31.0	0.2	1.0	2.9	0.3	21.6	0.1	40.8	145.8
Ecoprotector®	Average	83 ab	449 abc	3.7 ab	217 ab	3.4 abc	159 ab	31.0	242 abc	11.7 a	10.6	76 abc	1.6 ab	281 abc	2.1	659 ab	2230 abc
	Std Error	1.1	49.1	0.1	21.4	0.4	17.4	3.7	19.5	3.2	2.6	14.3	0.1	32.1	0.4	49.4	171.5
Envy	Average	58 ab	297 bc	3.1 ab	126 b	2.2 c	118 ab	27.4	160 abc	7.4 a	9.1	60 abc	1.2 ab	138 c	1.8	380 b	1389 bc
	Std Error	18.4	87.3	1.0	41.1	0.5	22.6	0.8	14.1	2.4	2.0	14.9	0.3	46.2	0.4	88.3	326.4
Fruit drying oil	Average	88 ab	897 a	4.4 ab	354 ab	3.9 abc	200 ab	35.9	305 abc	9.9 a	14.7	100 ab	1.7 ab	238 abc	4.7	869 ab	3126 abc
	Std Error	15.5	294.2	0.7	114.2	0.8	69.8	5.3	82.6	2.1	2.8	21.0	0.4	73.4	1.5	249.9	923.7
Parka Plus	Average	157 a	877 ab	8.2 a	433 a	5.9 ab	221 ab	36.4	352 a	11.7 a	12.2	105 ab	3.1 a	377 ab	6.3	1054 a	3660 a
	Std Error	37.6	107.7	2.2	43.4	1.0	37.7	4.2	32.9	1.3	1.3	14.8	1.1	25.9	0.9	184.4	397.6
Peratec	Average	79 ab	350 abc	3.8 ab	160 ab	2.9 abc	98 ab	23.4	172 abc	7.8 a	8.9	56 abc	1.8 ab	193 bc	3.5	521 ab	1681 abc
	Std Error	13.6	18.0	0.8	19.4	0.3	7.5	2.9	7.5	0.4	1.2	3.9	0.4	5.7	1.6	26.7	73.9
Raynox®	Average	117 ab	494 abc	6.0 ab	207 ab	4.3 abc	145 ab	31.7	230 abc	10.5 a	12.1	78 abc	2.3 ab	214 bc	3.1	636 ab	2191 abc
	Std Error	3.9	12.1	0.4	10.7	0.4	26.3	5.3	32.7	2.1	1.9	5.4	0.1	24.5	0.4	60.3	121.5
Silicone oil	Average	165 a	758 abc	8.0 a	348 ab	6.1 a	190 ab	32.7	307 abc	12.6 a	11.8	110 a	3.3 a	310 abc	4.6	940 ab	3207 ab
	Std Error	53.6	205.3	2.9	121.3	1.3	39.3	7.9	62.9	0.7	1.7	13.5	1.0	62.9	1.9	265.2	791.6
Surround®WP	Average	91 ab	473 abc	4.9 ab	213 ab	3.0 abc	153 ab	32.0	258 abc	6.6 a	9.4	61 abc	1.8 ab	180 c	3.5	565 ab	2055 abc
	Std Error	25.4	82.7	1.4	40.4	0.7	35.7	6.2	53.6	1.1	0.6	2.9	0.5	43.2	0.5	66.9	357.5
TiO_2[a]	Average	43 b	375 abc	2.1 ab	162 ab	1.8 c	112 ab	25.9	183 abc	8.8 a	10.2	63 abc	0.8 ab	166 c	2.1	426 ab	1582 bc
	Std Error	12.1	67.6	0.7	40.1	0.4	24.6	4.7	42.9	2.5	2.1	17.0	0.1	31.6	0.3	62.3	300.2
	P	0.004	0.001	0.010	0.002	<0.001	0.007	ns	0.002	0.069	ns	0.001	0.017	<0.001	ns	0.002	0.001

Values are means and standard errors of three replicates (n = 3) measured as syringol glucose-glucoside equivalents; Different letters within columns indicate statistically significant differences between treatment means (P = 0.05, one-way ANOVA); ns = not significant; Control (uncoated/no VP exposure) is not included in the statistical analysis and values listed are for a single replicate; Sy = syringol; Gu = guaiacol; Ph = phenol; Cr = cresol; GG = glucose-glucoside (gentiobioside); PG = pentose-glucoside; RG = rutinoside; MG = monoglucoside. [a]Applied in Raynox®; VP = volatile phenol.

Table S5. Concentrations of volatile phenols (μg/kg) in Shiraz grape homogenates after grapes were covered with various surface coatings and exposed to gaseous volatile phenols for 60 hours.

	Concentration (μg/kg)				
**Treatment**	**Guaiacol**	***o*-Cresol**	***m*-Cresol**	**Syringol**	**Total**
Control (uncoated/no VP exposure)	4	<1	<1	4	8
Control (uncoated/VP exposed)	13 ± 4.3	31 ± 9.3	7 ± 1.9	<2	51 ± 15.9
Biopest®	76 ± 36.7	165 ± 67.7	37 ± 15.0	6 ± 3.1	284 ± 121.9
Carbon[a]	121 ± 15.9	129 ± 20.3	37 ± 6.5	2.7 ± 1.5	290 ± 43.9
Deccoshield®	13 ± 4.1	33 ± 10.0	7 ± 1.8	<2	53 ± 16.5
Ecoprotector®	41 ± 5.2	101 ± 16.4	24 ± 4.7	4.3 ± 1.5	170 ± 27.2
Envy	18 ± 5.2	51 ± 17.5	10 ± 2.3	2 ± 0.9	81 ± 24.2
Fruit drying oil	160 ± 109.0	239 ± 135.3	55 ± 32.9	6.3 ± 3.4	460 ± 280.4
Parka Plus	92 ± 27.4	179 ± 44.8	41 ± 10.3	6 ± 0.6	318 ± 82.1
Peratec	36 ± 10.2	81 ± 15.6	19 ± 5.6	5 ± 2.0	141 ± 33.0
Raynox®	22 ± 3.9	54 ± 12.7	11 ± 2.9	3.7 ± 0.9	91 ± 19.9
Silicone oil	22 ± 5.5	53 ± 13.6	11 ± 2.3	3.3 ± 0.3	89 ± 21.1
Surround®WP	21 ± 5.8	46 ± 10.2	10 ± 2.1	2.7 ± 0.7	80 ± 18.6
Titanium dioxide[a]	30 ± 4.5	67 ± 6.0	15 ± 1.5	3.7 ± 0.3	116 ± 11.7
P	ns	ns	ns	ns	ns

Values are means ± standard error of three replicates (n = 3); Different letters within columns indicate statistically significant differences between treatment means ($P = 0.05$, one-way ANOVA); ns = not significant; Control (uncoated/no VP exposure) is not included in the statistical analysis and values listed are for a single replicate; [a]Applied in Raynox®; VP = volatile phenol.

Table S6. Concentrations of phenolic glycosides (µg/kg) in Shiraz grape homogenates after grapes were covered with various surface coatings and exposed to gaseous volatile phenols for 60 hours

		**Concentration (µg/kg SyGG equivalents)**															
**Treatment**		**SyGG**	**GuGG**	**PhGG**	**CrGG**	**SyPG**	**GuPG**	**PhPG**	**CrPG**	**GuRG**	**PhRG**	**CrRG**	**SyMG**	**GuMG**	**PhMG**	**CrMG**	**Total**
Control (uncoated/ no VP exposure)		<1	15	<1	<1	1	74	13	11	2	<1	5	<1	<1	<1	<1	121
Control (uncoated/ VP exposed)	Average	6.3 ab	360 d	1.0 ab	105 c	1.7	105 cd	33 a	197 bc	24 bc	21 ab	218 bc	0.4 cd	32 d	1.0	255 bc	1360 b
	Std Error	0.5	85.4	0.1	19.4	0.1	6.2	2.0	18.6	6.3	6.1	51.4	0.1	8.4	0.3	65.0	256.4
Biopest®	Average	40.3 a	1519 abc	2.9 ab	445 ab	2.1	184 bc	39 a	418 ab	65 abc	34 ab	466 ab	1.2 ab	146 abc	1.7	1051 a	4415 a
	Std Error	17.2	410.4	0.9	120.4	0.3	21.4	5.4	91.9	15.5	3.7	101.0	0.3	7.0	0.5	58.8	831.3
Carbon[a]	Average	0.5 b	141 d	0.5 b	37 c	1.4	76 d	26 a	77 c	12 c	15 b	88 c	0.2 d	11 d	0.7	92 c	578 b
	Std Error	0.4	17.7	0.0	6.2	0.1	6.2	1.4	3.5	2.3	3.1	19.2	0.03	1.5	0.3	15.6	62.3
Deccoshield®	Average	6.9 ab	483 cd	0.9 ab	166 bc	1.9	123 cd	34 a	245 bc	31 bc	27 ab	298 bc	0.4 cd	41 d	0.8	361 bc	1820 b
	Std Error	3.1	129.2	0.2	45.6	0.2	8.1	1.0	39.2	9.0	4.6	66.2	0.1	12.0	0.1	85.3	398.3
Ecoprotector®	Average	15.1 ab	697 bcd	1.4 ab	206 abc	1.7	139 bcd	36 a	302 bc	44 bc	30 ab	361 abc	0.7 abcd	63 c	1.2	495 bc	2393 b
	Std Error	6.1	55.0	0.3	22.3	0.2	2.3	1.7	25.3	3.6	3.1	30.9	0.2	14.9	0.5	105.4	249.3
Envy	Average	10.6 ab	482 cd	1.2 ab	138 c	1.8	120 cd	33 a	226 bc	31 bc	22 ab	241 bc	0.5 bcd	40 d	0.5	314 bc	1662 b
	Std Error	4.6	152.9	0.3	43.8	0.1	22.1	5.1	66.9	6.3	1.2	34.7	0.1	4.1	0.2	28.1	362.6
Fruit drying oil	Average	34.5 ab	2133 a	2.5 ab	468 a	2.2	300 a	42 a	600 a	115 a	36 a	656 a	0.9 abc	218 a	1.3	998 a	5607 a
	Std Error	12.2	498.4	0.6	90.6	0.3	23.0	6.8	80.6	24.9	4.9	111.6	0.2	50.1	0.4	166.4	1003.3
Parka Plus	Average	42.6 a	1773 ab	3.0 a	439 ab	2.3	215 b	34 a	443 ab	72 ab	26 ab	477 ab	1.4 a	159 ab	1.4	1014 a	4703 a
	Std Error	10.7	277.4	0.6	97.8	0.4	10.1	1.9	33.4	16.1	4.4	109.0	0.2	32.2	0.1	125.4	695.6
Peratec	Average	20.0 ab	814 bcd	1.6 ab	240 abc	1.9	151 bcd	34 a	308 bc	52 bc	31 ab	364 abc	0.7 abcd	74 bcd	1.0	527 b	2620 b
	Std Error	3.3	123.9	0.2	55.8	0.1	32.5	4.2	79.2	4.4	4.2	30.6	0.1	17.6	0.3	117.6	444.5
Raynox®	Average	12.2 ab	493 cd	1.2 ab	141 c	1.8	129 cd	34 a	255 bc	32 bc	25 ab	305 bc	0.5 bcd	47 d	0.8	380 bc	1858 b
	Std Error	1.8	99.5	0.1	22.7	0.1	7.2	5.9	51.1	2.3	2.9	26.7	0.1	7.9	0.2	14.1	195.7
Silicone oil	Average	13.0 ab	615 cd	1.3 ab	181 abc	1.6	147 bcd	43 a	283 bc	34 bc	24 ab	281 bc	0.4 cd	42 d	0.8	331 bc	1998 b
	Std Error	3.3	69.2	0.2	22.5	0.2	6.0	1.9	37.4	5.9	2.8	44.8	0.1	5.1	0.2	46.9	234.7
Surround®WP	Average	7.3 ab	451 cd	0.9 ab	133 c	1.7	110 cd	32 a	184 bc	24 bc	19 ab	229 bc	0.3 cd	34 d	0.6	285 bc	1512 b
	Std Error	0.5	22.1	0.1	9.2	0.2	11.7	3.5	24.6	1.1	0.7	7.9	0.1	2.1	0.1	9.5	40.4
TiO2[a]	Average	13.5 ab	673 cd	1.2 ab	184 abc	1.8	142 bcd	36 a	302 bc	38 bc	24 ab	313 bc	0.5 cd	61 cd	1.1	493 bc	2284 b
	Std Error	2.3	62.6	0.1	22.6	0.1	16.6	4.4	41.6	9.0	3.5	49.0	0.1	10.3	0.1	59.3	210.8
	P	0.003	<0.001	0.002	<0.001	ns	<0.001	ns	<0.001	<0.001	0.026	<0.001	<0.001	<0.001	ns	<0.001	<0.001

Values are means and standard errors of three replicates (n = 3) measured as syringol glucose-glucoside equivalents; Different letters within columns indicate statistically significant differences between treatment means ($P = 0.05$, one-way ANOVA); ns = not significant; Control (uncoated/no VP exposure) is not included in the statistical analysis and values listed are for a single replicate. Sy = syringol; Gu = guaiacol; Ph = phenol; Cr = cresol; GG = glucose-glucoside (gentiobioside); PG = pentose-glucoside; RG = rutinoside; MG = monoglucoside. [a]Applied in Raynox®; VP = volatile phenol.

Table S7. Correlations between the concentrations guaiacol, *o*-cresol, *p*-cresol, total volatile phenols and various aglycone groups of glycosides and total glycosides contained in Muscat Gordo and Shiraz grape homogenates after grapes were covered in various surface coatings and exposed to volatile phenols for 60 hours

	**guaiacol**	***o*-cresol**	***p*-cresol**	**total Sy glycosides**	**total Gu glycosides**	**total Ph glycosides**	**total Cr glycosides**	**total glycosides**
Muscat Gordo (Experiment 1)								
o-cresol	0.968							
p-cresol	0.972	0.981						
total Sy glycosides	0.521	0.619	0.657					
total Gu glycosides	0.737	0.832	0.771	0.685				
total Ph glycosides	0.525	0.562	0.548	0.709	0.737			
total Cr glycosides	0.718	0.806	0.758	0.763	0.964	0.838		
total glycosides	0.730	0.823	0.774	0.761	0.985	0.812	0.995	
total VPs	0.983	0.997	0.989	0.608	0.806	0.556	0.785	0.800
Muscat Gordo (Experiment 2)								
o-cresol	0.945							
p-cresol	0.944	0.993						
total Sy glycosides	0.288	0.426	0.449					
total Gu glycosides	0.653	0.748	0.733	0.757				
total Ph glycosides	0.400	0.521	0.527	0.792	0.814			
total Cr glycosides	0.582	0.704	0.701	0.845	0.976	0.875		
total glycosides	0.604	0.717	0.711	0.830	0.989	0.862	0.997	
total VPs	0.975	0.994	0.991	0.389	0.724	0.489	0.673	0.688
Shiraz								
o-cresol	0.975							
p-cresol	0.975	0.996						
total Sy glycosides	0.478	0.607	0.618					
total Gu glycosides	0.731	0.798	0.795	0.878				
total Ph glycosides	0.157	0.255	0.243	0.610	0.619			
total Cr glycosides	0.602	0.703	0.698	0.906	0.968	0.714		
total glycosides	0.671	0.756	0.752	0.901	0.992	0.675	0.992	
total VPs	0.990	0.997	0.995	0.566	0.777	0.218	0.669	0.728

Sy = syringol; Gu = guaiacol; Ph = phenol; Cr = cresol; Total Sy glycosides = sum of SyGG, SyPG and SyMG; Total Gu glycosides = sum of GuGG, GuPG, GuRG and GuMG; Total Ph glycosides = sum of PhGG, PhPG, PhRG and PhMG; Total Cr glycosides = CrGG, CrPG, CrRG and CrMG; Total glycosides = sum of all glycosides (n=15); GG = glucose-glucoside (gentiobioside); PG = pentose-glucoside; RG = rutinoside; MG = monoglucoside.

Table S8. Percentage compared to the control for the most abundant phenolic glycosides in Muscat Gordo grape homogenates after grapes were covered with various surface coatings and exposed to gaseous volatile phenols for 60 hours (Experiment 1)

	**% to the control**								
**Sample**	**SyGG**	**GuGG**	**CrGG**	**GuPG**	**PhPG**	**CrPG**	**CrRG**	**GuMG**	**CrMG**
Control (uncoated)	100	100	100	100	100	100	100	100	100
Biopest®	171	292	306	176	121	152	224	243	232
Carbon[a]	27	67	71	70	73	71	50	58	58
Deccoshield®	125	114	130	125	145	156	142	99	124
Ecoprotector®	225	227	248	146	151	159	197	142	181
Envy	100	102	98	86	98	91	97	63	87
Fruit drying oil	169	323	348	195	188	219	272	193	227
Parka Plus	197	214	296	135	111	153	184	167	179
Peratec	221	112	167	149	150	144	187	172	181
Raynox®	117	141	179	131	117	144	150	133	130
Silicone oil	141	124	137	118	139	127	148	137	130
Surround®WP	190	151	182	96	105	107	149	111	131
Titanium dioxide[a]	56	74	90	99	90	102	89	78	81

Values are means of three replicates (n = 3); Sy = syringol; Gu = guaiacol; Ph = phenol; Cr = cresol; GG = glucose-glucoside (gentiobioside); PG = pentose-glucoside; RG = rutinoside; MG = monoglucoside.
[a]Applied in Raynox®

Table S9. Percentage compared to the control for the most abundant phenolic glycosides in Muscat Gordo grape homogenates after grapes were covered with various surface coatings and exposed to gaseous volatile phenols for 60 hours (Experiment 2)

	% to the control								
**Sample**	**SyGG**	**GuGG**	**CrGG**	**GuPG**	**PhPG**	**CrPG**	**CrRG**	**GuMG**	**CrMG**
Control (uncoated)	100	100	100	100	100	100	100	100	100
Biopest®	176	239	274	315	185	262	195	364	280
Carbon[a]	38	75	93	95	104	83	67	119	100
Deccoshield®	163	131	167	177	170	186	130	149	152
Ecoprotector®	160	148	191	204	171	194	159	238	202
Envy	110	98	111	151	151	128	125	116	116
Fruit drying oil	168	294	311	256	198	245	208	201	266
Parka Plus	301	288	381	283	201	283	218	319	323
Peratec	151	115	141	125	129	138	117	163	159
Raynox®	224	162	182	186	175	185	162	181	195
Silicone oil	316	249	306	244	181	247	230	262	288
Surround®WP	175	155	187	197	176	207	127	152	173
Titanium dioxide[a]	82	123	143	144	143	147	131	141	130

Values are means of three replicates (n = 3); Sy = syringol; Gu = guaiacol; Ph = phenol; Cr = cresol; GG = glucose-glucoside (gentiobioside); PG = pentose-glucoside; RG = rutinoside; MG = monoglucoside.
[a]Applied in Raynox®

Table S10. Percentage compared to the control for the most abundant phenolic glycosides in Shiraz grape homogenates after grapes were covered with various surface coatings and exposed to gaseous volatile phenols for 60 hours

	% to the control								
**Sample**	**SyGG**	**GuGG**	**CrGG**	**GuPG**	**PhPG**	**CrPG**	**CrRG**	**GuMG**	**CrMG**
Control (uncoated)	100	100	100	100	100	100	100	100	100
Biopest®	636	422	423	176	117	212	214	460	412
Carbon[a]	8	39	36	72	77	39	40	34	36
Deccoshield®	109	134	158	117	103	125	137	129	141
Ecoprotector®	239	194	196	133	108	153	165	198	194
Envy	168	134	132	114	98	115	110	127	123
Fruit drying oil	545	593	445	286	125	305	300	688	391
Parka Plus	673	493	418	205	102	225	218	502	397
Peratec	315	226	228	144	101	157	167	233	207
Raynox®	193	137	134	123	102	129	140	148	149
Silicone oil	206	171	172	141	131	144	129	131	130
Surround®WP	115	125	126	105	97	93	105	107	111
Titanium dioxide[a]	213	187	175	136	109	153	143	193	193

Values are means of three replicates (n = 3); Sy = syringol; Gu = guaiacol; Ph = phenol; Cr = cresol; GG = glucose-glucoside (gentiobioside); PG = pentose-glucoside; RG = rutinoside; MG = monoglucoside.
[a]Applied in Raynox®
